# The Presentation and Treatment Response of Catatonia in Patients Admitted to the Psychiatric Inpatient Unit at Jimma University Medical Center, Ethiopia

**DOI:** 10.1155/2020/8739546

**Published:** 2020-06-30

**Authors:** Yimenu Yitayih, Elias Tesfaye, Kristina Adorjan

**Affiliations:** ^1^Department of Psychiatry, College of Health Sciences, Jimma University, Jimma, Ethiopia; ^2^Institute of Psychiatric Phenomics and Genomics (IPPG), Medical Center of the University of Munich, Germany; ^3^Department of Psychiatry and Psychotherapy, Medical Center of the University of Munich, Germany; ^4^Center for International Health, Ludwig-Maximilians-Universität, Munich, Germany

## Abstract

**Background:**

Catatonia is among the most mysterious and poorly understood neuropsychiatric syndrome. It is underresearched and virtually forgotten but still a frequent neuropsychiatric phenotype in both developed and low-income countries. Catatonia is associated with a number of medical complications like pulmonary embolism, dehydration, or pneumonia if it is not treated and managed adequately. In Ethiopia, however, almost no studies are available to describe the symptoms and the response to treatment in patients with catatonia. The aim of this retrospective study was therefore to describe the symptom profile of catatonia and to evaluate the treatment and outcome of catatonia in patients admitted to the psychiatric inpatient unit at Jimma University, Ethiopia.

**Method:**

Detailed treatment records of all inpatients were reviewed for the period from May 2018 to April 2019. All patients with catatonia at the inpatient unit of Jimma University Medical Center were assessed with the Bush-Francis Catatonia Rating Scale (BFCRS), and all comorbid psychiatric diagnoses were made according to the criteria of the Diagnostic Statistical Manual V. The presence and severity of catatonia were assessed by using the BFCRS at baseline and at discharge from the hospital.

**Result:**

In the course of one year, a total of 18 patients with the diagnosis of catatonia were admitted. The mean age of the participants was 22.8 years (SD 5.0; range: 15 to 34 years). The most common diagnosis associated with catatonia was schizophrenia (*n* = 12; 66.7%), followed by severe depressive disorders (*n* = 4; 22.2%). Mutism, posture, and withdrawal were registered in all patients (*n* = 18, 100%). All patients received an injection of diazepam and had improved at discharge.

**Conclusion:**

Our study provides further evidence that catatonia is most commonly associated with schizophrenia, followed by major depressive disorder, and that mutism, posturing, and withdrawal are the most common signs and symptoms of catatonia.

## 1. Background

Catatonia is a neuropsychiatric syndrome that is still poorly understood. Catatonia was first described by Kahlbaum as a state of psychomotor immobility and behavioral abnormality manifested by stupor, mutism, negativism, stereotypies, catalepsy, and verbigeration [1]. The prevalence of catatonia is 7-45% depending on the treatment setting [2–4]. Relevant studies have shown that up to 59% of patients with signs of catatonia are not recognized or underdiagnosed and 37% of these patients are not adequately treated [3]. The mortality rate in patients who are underdiagnosed with malignant catatonia, a particularly severe form, and are therefore not adequately treated is 75-100% in ICU settings [4, 5].

Patients with catatonia are unable to move normally, despite having a full physical capacity in the limbs and trunk [6]. The condition is characterized by a cluster of motor features, including mutism, a rigid posture, fixed staring, stereotypic movements, and stupor [7].

Catatonia is associated with many underlying psychiatric, neurologic, and medical disorders, including infections (such as encephalitis), autoimmune disorders, focal neurologic lesions (including strokes), metabolic disturbances, alcohol withdrawal, and abrupt or overly rapid benzodiazepine withdrawal [8–10]. The latest edition of the Diagnostic and Statistical Manual of Mental Disorders (DSM-5) of the American Psychiatric Association does not recognize catatonia as a separate disorder but describes it as related to another mental disorder or medical condition. Mental disorders that can be associated with catatonia include schizophrenia (catatonic type), bipolar disorder, posttraumatic stress disorder, and depressive disorders, as well as narcolepsy and drug abuse and overdose [11, 12]. The DSM-5 criteria for catatonia include the presence of three of the following twelve symptoms: stupor, catalepsy, waxy flexibility, mutism, negativism, posturing, mannerisms, stereotypy, agitation, grimacing, echolalia, and echopraxia. Other common symptoms include motor resistance to simple commands, posturing, rigidity, automatic obedience, and repetitive movements [11].

The underlying mechanisms of catatonia have remained widely obscure. The involvement of frontal lobe regions, lack of myelin protein, increased number of microglial cells is increasingly discussed [13–15]. A neuroinflammatory process in the area of the subcortical white matter, which may spread to the prefrontal cortex, is also assumed [16, 17]. Overall, it is assumed that catatonia is a dysregulation of the basal gangliothalamic-cortical control loop associated with motor, cognitive, sensory, and affective dysregulation and impaired impulse control [18].

Various neurotransmitter systems are also involved in the development of catatonia. A dopaminergic hypofunction with the typical symptoms of rigor and immobility, a decreased GABA-A activity, and a glutaminergic hypofunction associated with abnormal motor and social behavior patterns occur in catatonia [6, 19]. Since these neurotransmitter systems are also involved in other neuropsychiatric diseases and can cause similar symptom patterns, a thorough differential diagnostic classification is essential, especially with regard to delirium, autoimmune encephalopathy, neuroleptic malignant syndrome, serotonin syndrome, vegetative state, and neurodegenerative disorders [4, 20, 21].

Benzodiazepines and electroconvulsive therapy (ECT) are the most widely studied treatment methods. The response rate to benzodiazepines in Western studies lies between 66% and 100% [22–24]. ECT is a very effective therapy for catatonia, also when benzodiazepine (lorazepam) trials have failed [25–27]. The response percentages vary between 59% and 100% (26). Antipsychotics should be used with care as they can worsen catatonia and are the cause of neuroleptic malignant syndrome, a dangerous condition that can mimic catatonia and requires immediate discontinuation of the antipsychotic [28].

It is important to consider that catatonia is associated with a number of medical complications if not treated and managed adequately and that some of these complications are serious and life threatening. Pulmonary thromboembolism is a frequent cause of death in this patient group [4, 29].

However, in Ethiopia, studies are nonexistent in describing the sign and symptoms in patients presenting with catatonia. Furthermore, the treatment response and relation to sociodemographic and clinical variables of catatonia patients in Ethiopia have not yet been totally implicit. So, understanding catatonia is warranted in order to gain insight into signs/symptoms and the treatment outcome of catatonia in a given cultural context. Additionally, it will help to plan mental health services and policy, to organize training, to promote referrals to psychiatrists from other sources of health and social care, and to increase awareness by identifying the illness.

Therefore, the aim of this retrospective study was to describe the symptom profile of catatonia and to evaluate the treatment and outcome of catatonia among catatonic patients admitted to a psychiatric inpatient unit.

## 2. Materials and Methods

### 2.1. Study Design and Setting

This institution-based retrospective chart review study was conducted in inpatients at Jimma University Medical Center (JUMC), Southwest Ethiopia, from May 2018 to April 2019.

JUMC is in the city of Jimma, Oromia Region, in the southwest of Ethiopia, 352 km from Addis Ababa, the capital city. The hospital currently employs almost 1,000 people and each year provides tertiary care services for approximately 9,632 inpatients, 5,000 accident and emergency cases, and 80,000 outpatients from a catchment area population of 15 million. The psychiatry inpatient unit has 24 beds, which are mostly used for the management of acutely ill patients.

### 2.2. Participants

The study participants were patients with catatonia admitted to the JUMC psychiatric inpatient unit in the period of May 2018 to April 2019. The presence of a catatonia syndrome according to DSM-V was confirmed by the treating psychiatrist. The severity of the signs and symptoms of catatonia was assessed with the Bush-Francis Catatonia Rating Scale (BFCRS, see “Study Procedures” below). Treatment was started and managed by the treating psychiatrist/mental health specialist.

### 2.3. Study Procedures

Data were collected by five Master of Science in Psychiatry students. All data collectors were given training on the study objectives, data collection methods, and methods for maintaining confidentiality. The five students were supervised by two Master of Science in Public Health students and the principal investigator. On each data collection day, the completed questionnaires were checked for completeness. The data were entered into a computer and processed in a timely fashion. All patients with catatonia presenting to the JUMC inpatient unit were rated with the BFCRS, and all psychiatric diagnoses were made according to DSM-V criteria. The presence and severity of catatonia were assessed with the BFCRS at baseline and at discharge. The BFCRS has 23 items and is a widely used scale with established reliability and validity [30]. It serves to both screen for catatonia and estimate its severity. The first 14 items of the scale serve as a screening tool; a positive response to two of these screening items confirms the presence of catatonia. These 14 items and a set of nine additional items are used to rate the severity of the catatonia. In addition to the BFCRS, we used a questionnaire to assess the following variables of interest: socioeconomic factors (age, sex, marital status, ethnicity, religion, educational status, occupation, and income), treatment response (improved, worsened, death, and left the hospital against medical advice), type of medication taken, duration of illness, and length of stay in the hospital.

### 2.4. Data Processing and Analysis

After the tests and questionnaires had been checked for completeness, data were entered with EpiData Version 3.1 and then exported to Statistical Package for Social Sciences version 21.0 for further analysis. Descriptive statistics, such as the percentage and mean, were computed for the sociodemographic and clinical profile of patients. Catatonia symptoms and treatment responses were described by percentages.

## 3. Results

During the one-year period, a total of 18 patients were admitted with a diagnosis of catatonia (it includes catatonia due to schizophrenia, catatonia due to major depressive disorder, and catatonia due to bipolar disorder). The mean age of the participants was 22.8 years (SD 5.0; range: 15 to 34 years). There were more men (*n* = 14; 77.8%) than women (*n* = 4; 22.2%). Most of the patients were Oromo by ethnicity (*n* = 14; 77.8%) and from rural parts of the country (*n* = 12; 66.7%). Among the remaining sociodemographic variables, the most common were single marital status, primary level education, Muslim religion, and unemployed by occupation (see Table 1).

### 3.1. Clinical Profile of Participants

The most common diagnosis was catatonia associated with schizophrenia (*n* = 12; 66.7%), followed by catatonia associated with major depressive disorder (*n* = 4; 22.2%) and catatonia associated with bipolar disorder (*n* = 2; 11.1%). In 49% of patients, the onset of catatonia was associated with a precipitating event. The mean (SD) duration of catatonic symptoms at presentation was 82 (110) days (range: 11 to 365 days). In addition, the mean (SD) duration of catatonic symptoms for catatonia associated with schizophrenia was 115 (123) days; for major depressive disorder, 21 (10) days; and for bipolar disorder, 6.5 (1) days. A substance use disorder was present in 44.4% (*n* = 8) of patients, with khat use disorder being the most commonly abused substance.

### 3.2. Catatonic Signs/Symptoms as Rated on BFCRS

Mutism, posturing, and withdrawal were recorded in all patients (*n* = 18, 100%). The next most common signs and symptoms were staring (*n* = 16, 88.9%), immobility (*n* = 14, 77.8%), rigidity (*n* = 6, 33.3%), negativism (*n* = 4, 22.2%), waxy flexibility (*n* = 4, 22.2%), automatic obedience (*n* = 4, 22.2%), gegenhalten (*n* = 4, 22.2%), grimacing (*n* = 2, 11.1%), and mannerisms (*n* = 2, 11.1%) (Table 2). Waxy flexibility, rigidity, and grimacing were more common in the patients with schizophrenia than in those with major depressive disorder and bipolar disorder.

The mean (SD) length of hospital stay for all patients was 39.0 (19.8) days; for the patients with schizophrenia, 43.8 (21.1) days; for the patients with major depressive disorder, 36.5 (10.6) days; and for the patients with bipolar disorder, 15.0 (1.0) days. The mean (SD) BFCRS score of the patients at admission was 14 (2.5), and at discharge, 2.4 (2.2).

### 3.3. Management of Catatonia

First, all patients received an injection of diazepam. The mean (SD) time between administration of diazepam and starting psychotropic medication was 5.7 (4.8) days. Among the psychotropic medications, antipsychotics were the most commonly used (*n* = 14, 77.8%), followed by mood stabilizers (*n* = 4, 22.8%), and antidepressants (*n* = 2, 11.1%). Risperidone was the most commonly used antipsychotic (*n* = 12, 66.7%). All catatonic patients had improved at discharge, except for two patients who left against medical advice.

## 4. Discussion

In the current study, to describe the symptom profile of catatonia and treatment outcome, we reviewed the charts of inpatients presenting with catatonia. The most common catatonic signs and symptoms in this study were mutism, posturing, and withdrawal, all of which were present in all 18 patients. This finding is in keeping with previous research [3, 31]. In our study, most of the patients were younger than 30 years old and male. Some earlier studies reported that catatonia is more common in women [32], whereas others were in line with our study and found a greater proportion of men [33].

In this study, the most common mental disorder associated with catatonia was schizophrenia, followed by major depressive disorder. This finding is similar to that of a study performed in the United States of America [34, 35]; however, another study found that catatonia associated with mood disorder was more common than catatonia associated with schizophrenia [36]. In our study, there is no recorded catatonia sign of excitement, echopraxia/echolalia, stereotypy, mannerisms, verbigeration, stereotypy, impulsivity, grasp reflex, perseveration, and combativeness. It has also been argued that in comparison to classic psychiatric literature, modern research on catatonia has focused more on motor rather than verbal signs of catatonia, including echolalia and verbigeration [3, 37].

Our study suggests that there is a need for improved awareness on detecting verbal signs/symptoms of catatonia and that excited catatonia might be more frequently missed than retarded catatonia, knowing that excitement, echopraxia/echolalia, stereotypy, mannerisms, verbigeration, stereotypy, impulsivity, grasp reflex, perseveration, and combativeness are more frequently associated with excited catatonia [38].

In this study, the administration of diazepam relieved the symptoms of catatonia. Earlier studies demonstrated that benzodiazepines can relieve the signs and symptoms of catatonia in 70% of patients, and electroconvulsive therapy, in 85% of patients [39]. An earlier review found that benzodiazepines are only effective in 20% to 30% of cases of catatonic schizophrenia [40], but in this study, diazepam relieved catatonic signs and symptoms in 88.9% of the patients. In the case of acute-persistent catatonic symptoms, a detailed organ diagnosis and differential diagnosis should be performed in regard to autoimmune encephalitis and other organic causes of catatonia. Overall, a multidisciplinary treatment concept should be developed which may include a combination of different treatment approaches such as immune therapy, benzodiazepine, and ECT. Improving the detection and treatment of catatonia could help improve clinical outcomes of patients with this reversible syndrome in the general hospital.

Important limitations of this study are that it was a retrospective chart review with a small sample size. The study included only those patients who had been assessed with the BFCRS and were diagnosed as having catatonia according to DSM-V. The BFCRS has not been validated in Ethiopia. Future studies with larger sample sizes would possibly yield more important findings.

## 5. Conclusion

Our study provides further evidence that catatonia is most commonly associated with schizophrenia, followed by major depressive disorder, and that mutism, posturing, and withdrawal are the most common signs and symptoms of catatonia.

## Figures and Tables

**Table 1:** Sociodemographic characteristics of participants.

Sociodemographic variable		Whole sample, *n* (%)	Catatonia due to schizophrenia, *n* (%)	Catatonia due to major depressive disorder, *n* (%)	Catatonia due to bipolar disorder, *n* (%)
Age (y)	15-19	6 (33.3)	4 (66.7)	2 (33.3)	0 (0)
	20-24	4 (22.2)	2 (50)	0 (0)	2 (50)
	25-29	7 (38.9)	5 (71.4)	2 (28.6)	0 (0)
	30-34	1 (5.6)	1 (100)	0 (0)	0 (0)

Sex	Male	14 (77.8)	10 (71.4)	2 (14.3)	2 (14.3)
	Female	4 (22.2)	2 (50)	2 (50)	0 (0)

Marital status	Single	14 (77.8)	8 (57.1)	4 (28.6)	2 (14.3)
	Divorced/separated	4 (22.2)	4 (100)	0 (0)	0 (0)

Residential setting	Rural	12 (66.7)	8 (66.7)	4 (33.3)	0 (0)
	Urban	6 (33.3)	4 (66.7)	0 (0)	2 (33.3)

Educational status	No formal education	2 (11.1)	2 (100)	0 (0)	0 (0)
	Primary education	6 (33.3)	3 (50)	3 (50)	0 (0)
	Secondary education	5 (27.8)	4 (80)	1 (20)	0 (0)
	College and above	5 (27.8)	3 (60)	0 (0)	2 (40)

Occupational status	Unemployed	8 (44.4)	8 (100)	0 (0)	0 (0)
	Farmer	4 (22.2)	2 (50)	2 (50)	0 (0)
	Student	6 (33.3)	2 (33.3)	2 (33.3)	2 (33.3)

**Table 2:** Presence of symptoms on the Bush-Francis Catatonia Rating Scale in patients (*N* = 18) admitted to the psychiatric unit at Jimma University Medical Center.

Symptoms	Catatonic patients presenting symptom at admission, *n* (%)
Excitement	0 (0)
Immobility/stupor	14 (77.8)
Mutism	18 (100)
Staring	16 (88.9)
Posturing/catalepsy	18 (100)
Grimacing	2 (11.1)
Echopraxia/echolalia	0 (0)
Stereotypy	0 (0)
Mannerisms	2 (11.1)
Verbigeration	0 (0)
Rigidity	6 (33.3)
Negativism	4 (22.2)
Waxy flexibility	4 (22.2)
Withdrawal	18 (100)
Impulsivity	0 (0)
Automatic obedience	4 (22.2)
Mitgehen	1 (5.6)
Gegenhalten	1 (5.6)
Ambitendency	1 (5.6)
Grasp reflex	0 (0)
Perseveration	0 (0)
Combativeness	0 (0)
Autonomic abnormality	1 (5.6)

## Data Availability

Upon request, we can offer external researchers on-site access to the data at Jimma University, Ethiopia.

## References

[1] Khalbaum K. L. (1874). *Die Katatonie Oder das Spannungsirresein: Eine Klinische Form Psychischer Krankheit*.

[2] Stuivenga M., Morrens M. (2014). Prevalence of the catatonic syndrome in an acute inpatient sample. *Frontiers in Psychiatry*.

[3] Llesuy J. R., Medina M., Jacobson K. C., Cooper J. J. (2018). Catatonia under-diagnosis in the general hospital. *The Journal of Neuropsychiatry and Clinical Neurosciences*.

[4] Saddawi-Konefka D., Berg S. M., Nejad S. H., Bittner E. A. (2014). Catatonia in the ICU: an important and underdiagnosed cause of altered mental status. A case series and review of the literature. *Critical Care Medicine*.

[5] Daniels J. (2009). Catatonia: clinical aspects and neurobiological correlates. *The Journal of Neuropsychiatry and Clinical Neurosciences*.

[6] Madigand J., Lebain P., Callery G., Dollfus S. (2016). Syndrome catatonique : du depistage a la therapeutique. *L'Encéphale*.

[7] Fink M., Taylor M. A. (2009). The catatonia syndrome: forgotten but not gone. *Archives of General Psychiatry*.

[8] Rosebush P. I., Mazurek M. F. (1996). Catatonia after benzodiazepine withdrawal. *Journal of Clinical Psychopharmacology*.

[9] Deuschle M., Lederbogen F. (2001). Benzodiazepine withdrawal-induced catatonia. *Pharmacopsychiatry*.

[10] Lee J. W., Schwartz D. L., Hallmayer J. (2000). Catatonia in a psychiatric intensive care facility: incidence and response to benzodiazepines. *Annals of Clinical Psychiatry*.

[11] American Psychitaric Association (2013). *Diagnostic and Statistical Manual of Mental Disorders (DSM-5)*.

[12] Wilson J. E., Niu K., Nicolson S. E., Levine S. Z., Heckers S. (2015). The diagnostic criteria and structure of catatonia. *Schizophrenia Research*.

[13] Garcia-Agudo L. F., Janova H., Sendler L. E. (2019). Genetically induced brain inflammation byCnpdeletion transiently benefits from microglia depletion. *The FASEB Journal*.

[14] Janova H., Arinrad S., Balmuth E. (2018). Microglia ablation alleviates myelin-associated catatonic signs in mice. *Journal of Clinical Investigation*.

[15] Poggi G., Boretius S., Möbius W. (2016). Cortical network dysfunction caused by a subtle defect of myelination. *Glia*.

[16] Pease-Raissi S. E., Chan J. R. (2018). Micro (glial)-managing executive function: white matter inflammation drives catatonia. *The Journal of Clinical Investigation*.

[17] Rogers J. P., Pollak T. A., Blackman G., David A. S. (2019). Catatonia and the immune system: a review. *The Lancet Psychiatry*.

[18] Bakker P. R., Wichers M., van Harten P. N., Myin-Germeys I., Delespaul P., van Os J. (2013). Novel directions for psychiatric diagnosis: from psychopathology to motor function to monitoring technology. *Epidemiology and Psychiatric Sciences*.

[19] Margetić B., Aukst-Margetić B. (2011). Neuroleptic malignant syndrome and its controversies. *Pharmacoepidemiology and Drug Safety*.

[20] Barry H., Byrne S., Barrett E., Murphy K. C., Cotter D. R. (2015). Anti-N-methyl-d-aspartate receptor encephalitis: review of clinical presentation, diagnosis and treatment. *BJPsych Bulletin*.

[21] Mann A., Machado N. M., Liu N., Mazin A.-H., Silver K., Afzal K. I. (2012). A multidisciplinary approach to the treatment of anti-NMDA-receptor antibody encephalitis: a case and review of the literature. *The Journal of Neuropsychiatry and Clinical Neurosciences*.

[22] Yassa R., Iskandar H., Lalinec M., Cleto L. (1990). Lorazepam as an adjunct in the treatment of catatonic states. *Journal of Clinical Psychopharmacology*.

[23] Bush G., Fink M., Petrides G., Dowling F., Francis A. (1996). Catatonia. II. Treatment with lorazepam and electroconvulsive therapy. *Acta Psychiatrica Scandinavica*.

[24] Ungvari G. S., Leung C. M., Wong M. K., Lau J. (1994). Benzodiazepines in the treatment of catatonic syndrome. *Acta Psychiatrica Scandinavica*.

[25] Dutt A., Grover S., Chakrabarti S., Avasthi A., Kumar S. (2011). Phenomenology and treatment of catatonia: a descriptive study from north India. *Indian Journal of Psychiatry*.

[26] Raveendranathan D., Narayanaswamy J. C., Reddi S. V. (2012). Response rate of catatonia to electroconvulsive therapy and its clinical correlates. *European Archives of Psychiatry and Clinical Neuroscience*.

[27] Hatta K., Miyakawa K., Ota T., Usui C., Nakamura H., Arai H. (2007). Maximal response to electroconvulsive therapy for the treatment of catatonic symptoms. *The Journal of ECT*.

[28] Fink M., Taylor M. A. (2006). *Catatonia: A Clinician's Guide to Diagnosis and Treatment*.

[29] Gross A. F., Smith F. A., Stern T. A. (2008). Dread complications of catatonia: a case discussion and review of the literature. *Primary Care Companion to the Journal of Clinical Psychiatry*.

[30] Bush G., Fink M., Petrides G., Dowling F., Francis A. (1996). Catatonia. I. Rating scale and standardized examination. *Acta Psychiatrica Scandinavica*.

[31] Chalasani P., Healy D., Morriss R. (2005). Presentation and frequency of catatonia in new admissions to two acute psychiatric admission units in India and Wales. *Psychological Medicine*.

[32] Medda P., Toni C., Luchini F., Giorgi Mariani M., Mauri M., Perugi G. (2015). Catatonia in 26 patients with bipolar disorder: clinical features and response to electroconvulsive therapy. *Bipolar Disorders*.

[33] Kleinhaus K., Harlap S., Perrin M. C. (2012). Catatonic schizophrenia: a cohort prospective study. *Schizophrenia Bulletin*.

[34] Cohen D., Flament M., Dubos P. F., Basquin M. (1999). Case series: catatonic syndrome in young people. *Journal of the American Academy of Child & Adolescent Psychiatry*.

[35] Odayar K., Eloff I., Esterhuysen W. (2018). Clinical and demographic profile of catatonic patients who received electroconvulsive therapy in a South African setting. *South African Journal of Psychiatry*.

[36] van den Ameele S., Sabbe B., Morrens M. (2015). Characteristics of catatonia in schizophrenia and mood disorders. *Tijdschrift voor Psychiatrie*.

[37] Ungvari G. S., White E., Pang A. H. T. (2016). Psychopathology of catatonic speech disorders and the dilemma of catatonia: a selective review. *Australian & New Zealand Journal of Psychiatry*.

[38] Morrison J. R. (1973). Catatonia: retarded and excited types. *Archives of General Psychiatry*.

[39] Hawkins J. M., Archer K. J., Strakowski S. M., Keck P. E. (1996). Somatic treatment of catatonia. *The International Journal of Psychiatry in Medicine*.

[40] Rosebush P. I., Mazurek M. F. (2004). Pharmacotherapy of catatonia. *Catatonia: From Psychopathology to Neurobiology*.

